# Umbrella sampling and double decoupling data for methanol binding to *Candida antarctica* lipase B

**DOI:** 10.1016/j.dib.2021.107618

**Published:** 2021-11-22

**Authors:** Daniel Markthaler, Niels Hansen

**Affiliations:** University of Stuttgart, Institute of Thermodynamics and Thermal Process Engineering, Pfaffenwaldring 9, Stuttgart D-70569, Germany

**Keywords:** MD simulation, GROMACS, Umbrella sampling, Methanol, CALB

## Abstract

The binding free-energy profile of methanol to *Candida antarctica* lipase B (CALB) was calculated at infinite dilution and at a finite methanol concentration of 6.1 M using umbrella sampling molecular dynamics simulations with the OPLS all-atom force field. An additional validation of the results was performed by employing alchemical double decoupling simulations. The binding free-energy profiles have been used in a related research article to validate free-energy profiles obtained from direct counting simulations with the aim to use the kinetic information encoded in the latter. The data provided in this work will be useful to study concentration effects on binding, to test alternative free energy methods or to use the proposed simulation protocol for related systems.

**Specifications Table**

**Table:** 

Subject	Biological Sciences
Specific subject area	Computational Molecular Biophysics
Type of data	Molecular Dynamics (MD) Simulations, Figures
How data were acquired	Classical all-atom MD simulations in explicit solvent using GROMACS 2016.4
Data format	Raw: Time series of the COM-COM distance between protein and methanol for each umbrella window
	Analyzed: Free energy profiles and values
Parameters for data collection	OPLS all-atom force field
	NpT ensemble at 294 K
Description of data collection	Molecular dynamics simulations were conducted with GROMACS software, version 2016.4 patched to the free energy library PLUMED, version 2.4.2. Analysis was carried out with umbrella integration using an open source Python package (https://github.com/ATB-UQ/umbrella_integration) as well as with the open source Python package alchemical analysis (https://github.com/MobleyLab/alchemical-analysis).
Data source location	Institution: University of Stuttgart, Institute of Thermodynamics and Thermal Process Engineering
	City: Stuttgart
	Country: Germany
Data accessibility	Repository name: Data Repository of the University of Stuttgart (DaRUS)
	Data identification number: doi: 10.18419/darus-2104
Related research article	H. F. Carvalho, V. Ferrario, J. Pleiss, Molecular mechanism of methanol inhibition in CALB-catalyzed alcoholysis: Analyzing molecular dynamics simulations by a Markov state model, J. Chem. Theory Comput. 17 (2021) 6570-6582. https://doi.org/10.1021/acs.jctc.1c00559

## Value of the Data

• The data reported in this work can serve as reference for testing unbiased molecular dynamics simulations.
• The data will be useful for further investigation of the effect of concentration on binding free energy.
• The free energy profiles along with the input files and analysis scripts might be used to study related systems, to test alternative free energy approaches or to investigate other force fields.

## 1 Data Description

### 1.1 Free-energy profiles

**Fig. 1:**
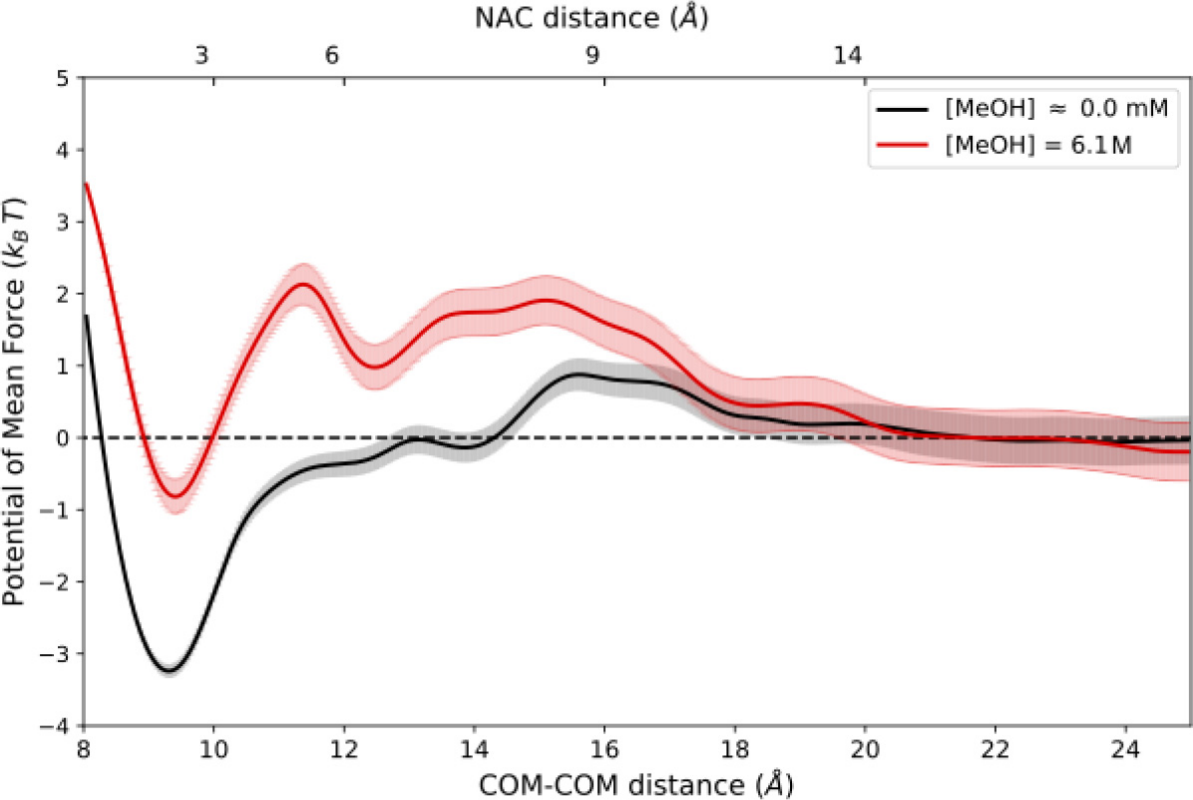
Potentials of mean force (in $k_{B}T$ units) as function of the COM-COM distance, $d_{\mathrm{COM}}$ (bottom axis) and $d_{\mathrm{NAC}}$ (top axis) between ^acyl^CALB and methanol at infinite dilution (≈0 M) and 6.1 M concentrations, obtained from umbrella sampling. Reported error bounds corresponding to the applied umbrella integration method represent cumulative estimates over the considered range of $d_{\mathrm{COM}}$ values [8].

Potentials of mean force (PMFs) for methanol binding to an acyl intermediate of lipase B of *C. antarctica*, referred to as ^acyl^CALB, were calculated from umbrella sampling (US) simulations at infinite dilution and at 6.1 M methanol concentration (Fig. 1). The raw data for Fig. 1 is provided in the dataset accompanying this work, referred to in the ‘Data accessibility’ section in the ‘Specifications Table’. It can be accessed via unpacking the archive ‘UmbrellaSampling.tar.gz’ and following the directory tree towards the folder ‘results’. The restrained coordinate was the distance between the centers of mass ($d_{\mathrm{COM}}$) of protein and methanol. For distances $d_{\mathrm{COM}}>20$ Å, the PMFs become flat (as expected for two non-interacting particles), indicating that the substrate is in the bulk and can therefore be considered as being unbound. The secondary x-axis in Fig. 1 corresponds to an alternative distance definition, in which the distance toward a productive binding pose of the substrate is measured, referred to as near attack conformation (NAC). This definition was used by Carvalho et al. [4] in the related research article to analyze unbiased molecular dynamics simulations, performed for the same system, by a Markov state model.

The PMFs for both concentrations feature a global minimum at $d_{\mathrm{COM}}\approx 9$ Å. In the limiting case of infinite dilution, the free energy difference between the bound and unbound state is $-3k_{B}T$. At 6.1 M, this free energy difference increased to $-1k_{B}T$. This significant decrease in binding affinity with increasing methanol concentration is attributed to the bulk-like microenvironment in the binding site. The 6.1 M profile shows local barriers at $d_{\mathrm{COM}}\approx 11$ Å  ($d_{\mathrm{NAC}}\approx 5$ Å) and $d_{\mathrm{COM}}\approx 16$ Å  ($d_{\mathrm{NAC}}\approx 9$ Å), whereas at infinite dilution only the latter barrier is present.

### 1.2 Double decoupling

**Fig. 2:**
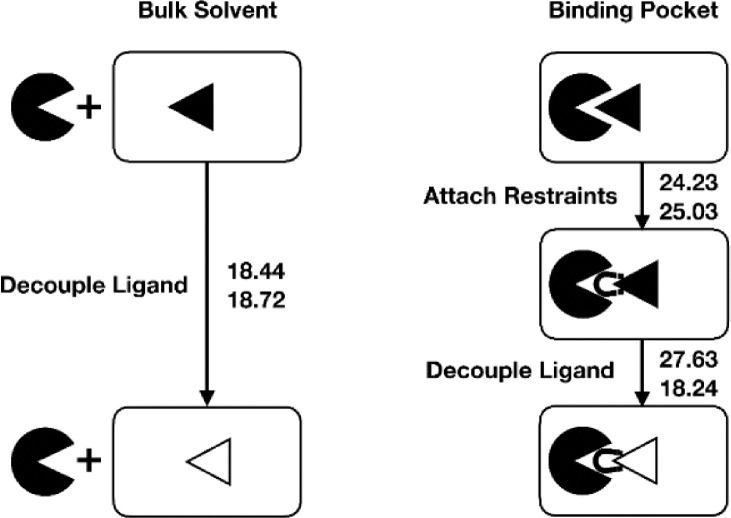
Double decoupling method applied to ^acyl^CALB-methanol. The filled (open) triangle represents the fully interacting (decoupled) ligand. The left branch corresponds to the decoupling of the methanol ligand in the bulk solvent (i.e. the unbound state), while the reverse process would correspond to the hydration of one methanol molecule. The right branch corresponds to the decoupling of the methanol ligand inside the binding pocket of the protein (i.e. the bound state), including the preceding activation of translational/orientational restraints in order to prevent diffusion of the decoupled ligand. Reported numerical values represent the free energy estimates (in $\mathrm{kJ}\;{\text{mol}}^{-1}$) according to the MBAR estimator [9], [12]. The upper (lower) value corresponds to the methanol concentration of infinite dilution (6.1 M).

The raw data for Fig. 2 is provided in the dataset accompanying this work, referred to in the ‘Data accessibility’ section in the ‘Specifications Table’. It can be accessed via unpacking the archive ‘DoubleDecoupling.tar.gz’ and following the directory tree towards the folders ‘dhdl_data_mbar’ which are present for each branch shown in Fig. 2. The decoupling (or negative solvation) free energies of methanol in the bulk solvent were found to be nearly independent of the methanol concentration, as judged by the very similar estimates at infinite dilution and at 6.1 M (Fig. 2). Comparison with experimental estimates for the hydration free energy of methanol at $25^{\circ }$C ($-21.4\;\mathrm{kJ}\;{\text{mol}}^{-1}$) [3] and the methanol self-solvation free energy at $25^{\circ }$ ($-20.3\;\mathrm{kJ}\;{\text{mol}}^{-1}$) [11] shows reasonable agreement and confirms the observed independence of the methanol concentration. The decoupling free energies in the protein binding pocket in contrast, revealed a considerable dependence on the methanol concentration. While the decoupling free energy at 6.1 M ($18.2\;\mathrm{kJ}\;{\text{mol}}^{-1}$) was very close to the corresponding value in the bulk, the value at infinite dilution was significantly higher ($27.6\;\mathrm{kJ}\;{\text{mol}}^{-1}$). The free energy cost for restraining the translational and orientational movement of the interacting methanol ligand in the binding pocket was also found to be almost concentration independent. The high values of this free energy change can be attributed to the penalty for restraining the orientational movement of the ligand. Comparison of the difference depicted in Fig. 2 reveals that the major decrease in the binding affinity at high methanol concentration arises from the change in the decoupling free energy in the binding pocket. This change in the decoupling free energy ((18.2+25.0)−(27.6+24.2) = $-8.6\;\mathrm{kJ}\;{\text{mol}}^{-1}$ ≈ $-3.5\;k_{B}T$) is in reasonable agreement with the difference in the global PMF minima of the corresponding concentrations in Fig. 1, which indicates the robustness of the free energy profiles obtained in the present work.

## 2 Experimental Design, Materials and Methods

### 2.1 Umbrella sampling

All input, coordinate and topology files required to reproduce the US simulations are provided in the dataset accompanying this work, referred to in the ‘Data accessibility’ section in the ‘Specifications Table’. Umbrella sampling (US) was performed to explore binding of methanol to ^acyl^CALB along the radial distance $d_{\mathrm{COM}}$ between the centers-of-mass (COM) of the protein and substrate molecule. A set of 35 umbrella windows were equally distributed between $d_{\mathrm{COM}}=8$ and 25 Å, using harmonic distance restraints with a force constant of $3000\;\mathrm{kJ}\;{\text{mol}}^{-1}{\text{nm}}^{-2}$. US simulations were conducted with the GROMACS MD code (version 2016.4) [1] patched to the free-energy library PLUMED (version 2.4.2) [14] for restraints handling. To enhance configurational sampling at each umbrella window, Hamiltonian replica exchange [13] was applied to enable the exchange of Hamiltonians between neighboring windows. The difference of the Hamiltonians is defined by the individual centers of the distance restraining potential. An exchange was attempted every 1000 steps and accepted based on the Metropolis-Hastings criterion. Initial configurations for each window were generated within a sequence of prior short simulations (200 ps per window) by gradually displacing the ligand out of the binding pocket into the bulk, starting from an equilibrated configuration with the selected ligand bound to the protein. Two concentrations were considered: infinite dilution and 6.1 M. The latter concentration was calculated based on the mean simulation box volume from which an estimate of the protein volume [5] was subtracted. The systems were simulated for 60 ns per window, until stable free energy profiles were obtained. For consistency with the corresponding unbiased simulations  [4], identical simulation parameters and system sizes were employed. Free energy profiles were estimated using the umbrella integration method [8]. Estimation based on alternative analysis methods such as the weighted histogram analysis method (WHAM) [6], [7] and the MBAR estimator [12] yielded identical free energy profiles within statistical uncertainties. Independence of the profiles from the used window spacing was verified during post-processing by considering only the sampled data from every second or third window. The free-energy profiles were converted to potentials of mean force by removing the Jacobian contribution of $2k_{B}T\ln\left(d_{\mathrm{COM}}\right)$ [15]. The NAC distance was defined as $d_{\mathrm{NAC}}=\sqrt{0.5(d_{1}^{2}+d_{2}^{2})}$, where $d_{1}$ corresponds to the distance between the hydroxyl oxygen of methanol and the carbonyl carbon of ^acyl^Ser105 and $d_{2}$ to the distance between the hydroxyl hydrogen of methanol and the nitrogen of His224.

### 2.2 Double Decoupling

All input, coordinate and topology files required to reproduce the double decoupling simulations as well as the simulation output leading to the values reported in Fig. 2 are provided in the dataset accompanying this work, referred to in the ‘Data accessibility’ section in the ‘Specifications Table’. The free energy profiles obtained from the US simulations were further validated [10] by applying the double decoupling method to the system ^acyl^CALB-methanol for the two concentrations (infinite dilution and 6.1 M). From the separate alchemical decoupling of the ligand (i.e. a single specified methanol molecule) in the bulk solvent and in the binding pocket, the impact of finite substrate concentrations on the difference between free energy profiles was analyzed. For the bulk simulations, cubic boxes with binary mixtures of compositions (1 methanol/1281 water molecules) for infinite dilution and (146 methanol/1000 water) for the 6.1 M concentration were considered. For decoupling simulations of the ligand inside the binding pocket, identical system sizes and simulation parameters as in the US simulations were employed. The scaling of the non-bonded interactions between the ligand and its environment was controlled via a coupling parameter λ, such that λ=0 and λ=1 represents the fully interacting and fully decoupled ligand, respectively, while retaining the intramolecular interactions. The decoupling was conducted in a sequence of 20 discrete steps, using simulation times of 20 ns per λ-state. In the applied perturbation scheme, electrostatic interactions were deactivated first within 5 steps, followed by the deactivation of the Lennard-Jones interactions. To avoid numerical problems close to the end states, soft-core (sc) potentials were used with parameters $\alpha_{\mathrm{sc}}=0.5$, $\sigma_{sc}=0.3$ nm and a power for the soft-core scaling function of $p_{\mathrm{sc}}$ = 1 [1]. For decoupling of methanol from the protein in case of the finite concentration, sampling was enhanced through the usage of Hamiltonian replica exchange, enabling the Hamiltonians of neighboring lambda points to swap every 1000 steps with a probability based on the Metropolis-Hastings criterion. Therefore, initial configurations for every lambda point were generated within a short prior stratification simulation without exchanges between lambda points. To prevent translational and orientational diffusion of the decoupled methanol ligand in the binding pocket, a set of six harmonic restraints, comprising one restrained distance ($r_{\mathrm{aA}}$ = dist(a,A)), two angles ($\theta_{A}$ = angle(b,a,A), $\theta_{B}$ = angle(a,A,B)) and three dihedral angles ($\phi_{A}$ = dihed(c,b,a,A), $\phi_{B}$ = dihed(b,a,A,B), $\phi_{C}$ = dihed(a,A,B,C)) were imposed for the protein-ligand complex [2]. Therefore, a set of anchor atoms in the protein (a: C819^Ser105^, b: NE2^His224^, c: H^Thr40^) and in the ligand (A: O, B: HO, C: C) were selected [2]. Reference values for the restraining potentials were estimated from a preceding unbiased simulation with the ligand bound to the binding pocket, while the corresponding force constants were set to $500\;\mathrm{kJ}\;{\text{mol}}^{-1}{\text{nm}}^{-2}$ for the restrained distance $r_{\mathrm{aA}}$ and $50\;\mathrm{kJ}\;{\text{mol}}^{-1}{\text{rad}}^{-2}$ for all (dihedral) angles. For the chosen reference value for $r_{\mathrm{aA}}$, the COM-COM distance $d_{\mathrm{COM}}$ between protein and ligand is close to the PMF minimum (Fig. 1). Since the free energy cost for activating these auxiliary restraints is only weakly influenced by the (bulk) substrate concentration, identical restraints specifications have been used for both considered concentrations. This free energy contribution was evaluated from simulations of the bound and fully interacting ligand within 8 distinct steps by uniformly increasing the force constants between 0 and the final values reported above. The difference between the free energy contributions in the unbound state and the bound state is concentration dependent and reflects the difference between the free energy profiles in the binding site. All free energy changes (decoupling, activation of restraints) were estimated from the sampled potential energy differences between all λ-states using the MBAR estimator [12] as implemented in a freely available Python program [9].

## Ethics Statement

This work meets the ethical requirements for publication (https://www.elsevier.com/authors/journal-authors/policies-and-ethics) and did not involve studies with humans or animals.

## CRediT Author Statement

**Daniel Markthaler:** Investigation, Formal analysis, Writing - original draft; **Niels Hansen:** Conceptualization, Supervision, Writing – original draft, Project administration.

## Declaration of Competing Interest

The authors declare that they have no known competing financial interests or personal relationships which have, or could be perceived to have, influenced the work reported in this article.

## References

[1] Abraham M.J., Murtola T., Schulz R., Páll S., Smith J.C., Hess B., Lindahl E. (2015). GROMACS: High performance molecular simulations through multi-level parallelism from laptops to supercomputers. SoftwareX.

[2] Boresch S., Tettinger F., Leitgeb M., Karplus M. (2003). Absolute binding free energies: A quantitative approach for their calculation. J. Phys. Chem. B.

[3] Cabani S., Gianni P., Mollica V., Lepori L. (1981). Group contributions to the thermodynamic properties of non-ionic organic solutes in dilute aqueous solution. J. Solution Chem..

[4] Carvalho H.F., Ferrario V., Pleiss J. (2021). Molecular mechanism of methanol inhibition in CALB-catalyzed alcoholysis: Analyzing molecular dynamics simulations by a Markov state model. J. Chem. Theory Comput..

[5] Chen C.R., Makhatadze G.I. (2015). ProteinVolume: Calculating molecular van der Waals and void volumes in proteins. BMC Bioinf..

[6] Ferrenberg A.M., Swendsen R.H. (1989). Optimized Monte Carlo data analysis. Phys. Rev. Lett..

[7] Hub J.S., de Groot B.L., van der Spoel D. (2010). g_wham - a free weighted histogram analysis implementation including robust error and autocorrelation estimates. J. Chem. Theory Comput..

[8] Kästner J., Thiel W. (2006). Analysis of the statistical error in umbrella sampling simulations by umbrella integration. J. Chem. Phys..

[9] Klimovich P.V., Shirts M.R., Mobley D.L. (2015). Guidelines for the analysis for free energy calculations. J. Comput. Aided Mol. Des..

[10] Luzhkov V.B. (2008). Calculation of PMF from the WHAM and FEP molecular dynamics simulations: Case study of the methane dimer in water. Chem. Phys. Lett..

[11] Moine E., Privat R., Sirjean B., Jaubert J.N. (2017). Estimation of solvation quantities from experimental thermodynamic data: Development of the comprehensive CompSol databank for pure and mixed solutes. J. Phys. Chem. Ref. Data.

[12] Shirts M.R., Chodera J.D. (2008). Statistically optimal analysis of samples from multiple equilibrium states. J. Chem. Phys..

[13] Sugita Y., Kitao A., Okamoto Y. (2000). Multidimensional replica-exchange method for free-energy calculations. J. Chem. Phys..

[14] Tribello G.A., Bonomi M., Branduardi D., Camilloni C., Bussi G. (2014). PLUMED 2: New feathers for an old bird. Comput. Phys. Commun..

[15] Trzesniak D., Kunz A.-P.E., van Gunsteren W.F. (2007). A comparison of methods to compute the potential of mean force. ChemPhysChem.

